# Carbon nanomaterial hybrids via laser writing for high-performance non-enzymatic electrochemical sensors: a critical review

**DOI:** 10.1007/s00216-021-03382-9

**Published:** 2021-05-12

**Authors:** Marcel Simsek, Nongnoot Wongkaew

**Affiliations:** Institute of Analytical Chemistry, Chemo- and Biosensors, University of Regensburg, 93053 Regensburg, Germany

**Keywords:** Non-enzymatic sensor, Electrochemical detection, Nanocatalysts, Carbon nanomaterials, Laser-induced carbon

## Abstract

Non-enzymatic electrochemical sensors possess superior stability and affordability in comparison to natural enzyme-based counterparts. A large variety of nanomaterials have been introduced as enzyme mimicking with appreciable sensitivity and detection limit for various analytes of which glucose and H_2_O_2_ have been mostly investigated. The nanomaterials made from noble metal, non-noble metal, and metal composites, as well as carbon and their derivatives in various architectures, have been extensively proposed over the past years. Three-dimensional (3D) transducers especially realized from the hybrids of carbon nanomaterials either with metal-based nanocatalysts or heteroatom dopants are favorable owing to low cost, good electrical conductivity, and stability. In this critical review, we evaluate the current strategies to create such nanomaterials to serve as non-enzymatic transducers. Laser writing has emerged as a powerful tool for the next generation of devices owing to their low cost and resultant remarkable performance that are highly attractive to non-enzymatic transducers. So far, only few works have been reported, but in the coming years, more and more research on this topic is foreseeable.

**Graphical abstract:**

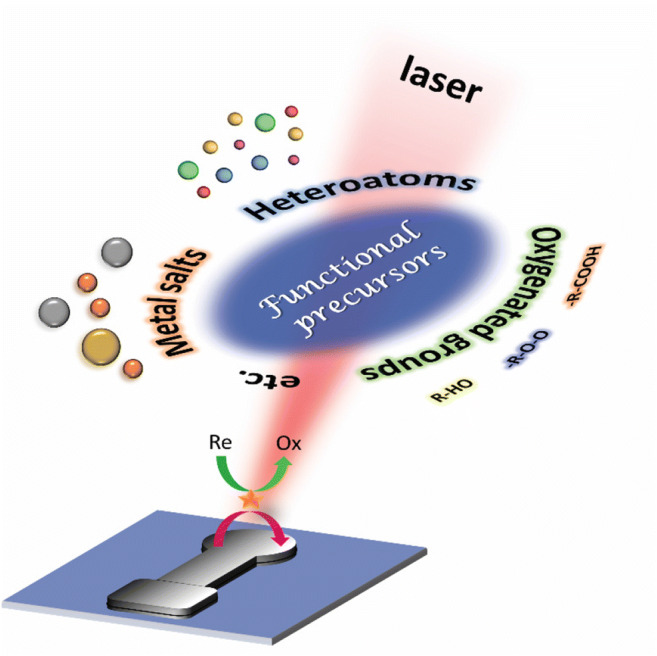

## Introduction

The next generation of analytical devices should not only offer superior sensing performance for analytes of interest but also be feasibly massively produced in a reliable and cost-effective manner, especially in healthcare applications where point-of-care testing (POCT), wearable sensors, and cell-based assays have been envisioned for future developments [1]. Electrochemical sensors offer beneficial features such as miniaturized system capability along with improved sensing performance, fast response time, high affordability, and instrumental portability, making them an outstanding strategy among others for advancing the fields. Electrochemical enzyme-based sensors with high selectivity and sensitivity have long been proposed and successfully commercialized, e.g., glucometer for monitoring blood glucose of diabetes patients. Typically, a redox enzyme, e.g., oxidase or reductase, is immobilized on an electrochemical transducer. A conversion of an analyte (as a substrate) to a product by the enzyme leads to changes that can be consequently monitored by the electrode held at a proper potential. The first generation of these sensors indirectly monitored the analyte concentration via the change of a co-substrate, e.g., O_2_, or a co-product, e.g., H_2_O_2_, produced by the enzymatic reaction. However, dependency on the uncontrollable level of dissolved O_2_ and the requirement of a high electrochemical potential to detect the redox indicators greatly decrease the reliability of sensing performance. Thus, the second sensor generation was developed by using an artificial redox mediator to purposely shuttle electrons from the active site of the enzyme to the electrode surface where a relatively low potential is commonly required for the mediator. Further improvement in the third generation lies in the ability to directly monitor electron transfer between enzyme and electrode. This can be realized, for example, by the aid of nanomaterial wiring between cofactor and electrode surface [2]. These enzyme-based electrochemical sensors have demonstrated their potential in a wide range of applications including for POCT and wearable devices. Even though enzyme-based electrochemical sensors have proven their remarkable selectivity and sensitivity over decades, they still suffer from the inherent consequences of using a complex biological agent. These drawbacks include poor stability, high production cost, high complexity of fabrication methods, and strict storage conditions, and because of those, applications in long-term uses and in resource-limiting areas are hindered.

In contrast, non-enzymatic electrochemical sensors, the fourth generation, take advantage of electrode capability in promoting electrocatalytic reaction instead of using enzymes. Thus, those aforementioned issues connected to the use of enzymes can be overcome. The comparison of enzymatic vs. non-enzymatic approaches shown in Table 1 elucidates why non-enzymatic sensors are a hot research topic nowadays. Research activities on this topic, in particular towards glucose detection, have been actively demonstrated since 2013 with the obvious rising of publication numbers up to 3–4 times in comparison to the previous years. A wide variety of nanomaterials have been explored for facilitating electrocatalytic reactions with an appreciable sensitivity for various analytes highly comparable to natural enzymatic counterparts. Nanomaterials, including transition metals, metal oxides (or sulfides), metal composites (e.g., alloy), and carbon and its derivatives, as well as hybrids of carbon and metals, are reported as potential catalysts for promoting electrocatalytic activity in the non-enzymatic electrochemical sensors. In principle, these nanomaterials enable electrocatalytic reaction by facilitating the adsorption between analyte and electrochemical interface where subsequent redox reaction can take place in an efficient manner. As a result, a considerable low reduction or oxidation potential is required to generate electrochemical signals.

**Table 1:** Evaluation of pros and cons for enzymatic and non-enzymatic electrochemical sensors

	Enzymatic	Non-enzymatic
Advantages	• Highly selective • Highly sensitive • Good biocompatibility • Commonly operating under physiological conditions	• Highly sensitive • Broad variety of nanomaterials available at low costs • Simple fabrication and compatible to mass production • Rather stable under sterilization conditions • Storage under ambient conditions feasible • Good long-term stability • Good biocompatibility for some nanocatalysts, e.g., noble metal nanoparticles
Disadvantages	• High material costs • High complexity in fabrication process • Low production throughput • Unstable under sterilization • Requirement of specific storage condition • Short-term stability	• Low selectivity • Some nanocatalysts require alkaline media for electrocatalytic reaction

Nowadays, hybrids made of carbon nanomaterials and metal nanocatalysts or heteroatoms have attracted a great deal of attention due to their relatively low cost, high stability, and good electrical conductivity. Many carbon nanomaterials, especially in the form of 3-dimensional (3D) architectures, have been proposed as high-performance non-enzymatic transducers owing to their immense surface area providing a high number of active sites. Most non-enzymatic electrochemical sensors developed have in fact achieved favorable analytical sensitivity for the target analyte. However, their translation into real-world applications has not been seen in the last decade, so far. One major obstacle is likely the fact that currently proposed fabrication methods for those nanomaterials are too complex and do not lend themselves for scaling-up or integrating into miniaturized devices. Many of the fabricated non-enzymatic-based sensors with reported outstanding performance were relied on conventional macroelectrode supports, e.g., glassy carbon electrodes (GCEs), using binders for integration. However, once the methods and sensing designs are further used in large-scale production, analytical performance and cost remain the crucial questions if they behave as good as when they do on the GCEs, for instance.

Various practical approaches have been proposed to create 3D-non-enzymatic transducers. This review, however, focuses on the generation of functional carbon nanomaterials and hybrids via laser writing. In the literature, the term “laser writing” can be seen interchangeably as laser induction, laser exposure, laser scribing, laser engraving, or laser irradiation which has emerged as a cost-effective means to fabrication. A wide variety of functional carbon nanomaterials with electrocatalytic activities have been generated through laser writing, paving the way to create non-enzymatic electrochemical transducers with high flexibility in electrode designs and choice of substrates. In comparison to conventional screen-printing techniques or other-based lithography, this strategy is considered more attractive for the following reasons: (1) rapid prototyping without the need of a block screen to be initially generated; (2) preserved beneficial features of 3D-porous structure after electrode generation; (3) freedom from binders; and (4) simple instrumentation and operating systems. However, to the best of our knowledge, the strategy of laser writing has not been employed extensively in generating non-enzymatic electrochemical sensors [3]. Recently, a review article by Han et al. [4] has pointed out the potential of laser in printing flexible sensors with a focus on resistive and capacitive-based sensors. In addition, the advances in electrochemical sensors and biosensors realized by using laser-derived graphene have been comprehensively reviewed [5]. These review articles have suggested a significant growth in the use of the laser-assisted strategy in generating analytical devices. However, none of these articles discuss exploiting the strategy for non-enzymatic electrochemical sensors. Therefore, this critical review aims to evaluate opportunities and merits of using laser to generate functional transducers for high-performance enzyme-free sensors. We will first give a brief overview of functional nanomaterials that serve as catalysts in non-enzymatic electrochemical sensors and their reaction mechanisms to bring readers into the field. Then, we will discuss the existing laser writing technology and its feasibility to generate a variety of functional carbon nanomaterials and hybrids that have been exploited in non-enzymatic sensors as well as those proposed for other applicable areas, e.g., energy storage. The discussion will include their merits over traditional strategies and factors that govern successful fabrication. Furthermore, challenges remaining in the use of laser-induced non-enzymatic transducers will be discussed to guide future prospects for research and developments.

## Non-enzymatic electrochemical sensor

### Electrocatalytic sites and mechanisms

Non-enzymatic (or enzyme-free) electrochemical sensors rely, in principle, on the reaction of an analyte catalyzed by the modified electrode or electrode material itself which facilitates fast electron transfer kinetics at a lower potential than that of a normal electrode material. For instance, the oxidation of glucose at GCE in alkaline media required the potential of approx. 1.0 to 1.4 V to yield a prominent anodic peak [6], while the potential at ca. 0.2–0.6 V (depending on the type of catalyst) was suitable with most non-enzymatic transducers [7]. A wide variety of electrocatalysts for non-enzymatic sensors have been investigated, which include metals, metal composites, metal oxides, nanocarbon, and its derivatives which will be elaborated in the next section. Even though the emphasis will be put more on carbon nanomaterials, brief discussion will be also given to traditional metal-based electrocatalysts as its fundamental knowledge for the hybrid carbon catalysts.

For transition metals, the electrocatalytic process takes place via the adsorption of the analyte onto the electrode surface and subsequent breaking of a bond where the product is later undergone desorption [8]. Among many analyte species, glucose has been dominantly investigated via two proposed electrocatalytic model mechanisms. The first model relies principally on activated chemisorption as suggested by Pletcher [9]. This model, nevertheless, does not account for the oxidative role of hydroxyl radicals that are largely evident in many studies [7] where the electrooxidation of glucose and other organic molecules can take place at adsorbed hydroxyl groups (OH_ads_). As a result, Burke [10] proposed the “Incipient Hydrous Oxide Adatom Mediator” model (IHOAM).

**Fig. 1:**
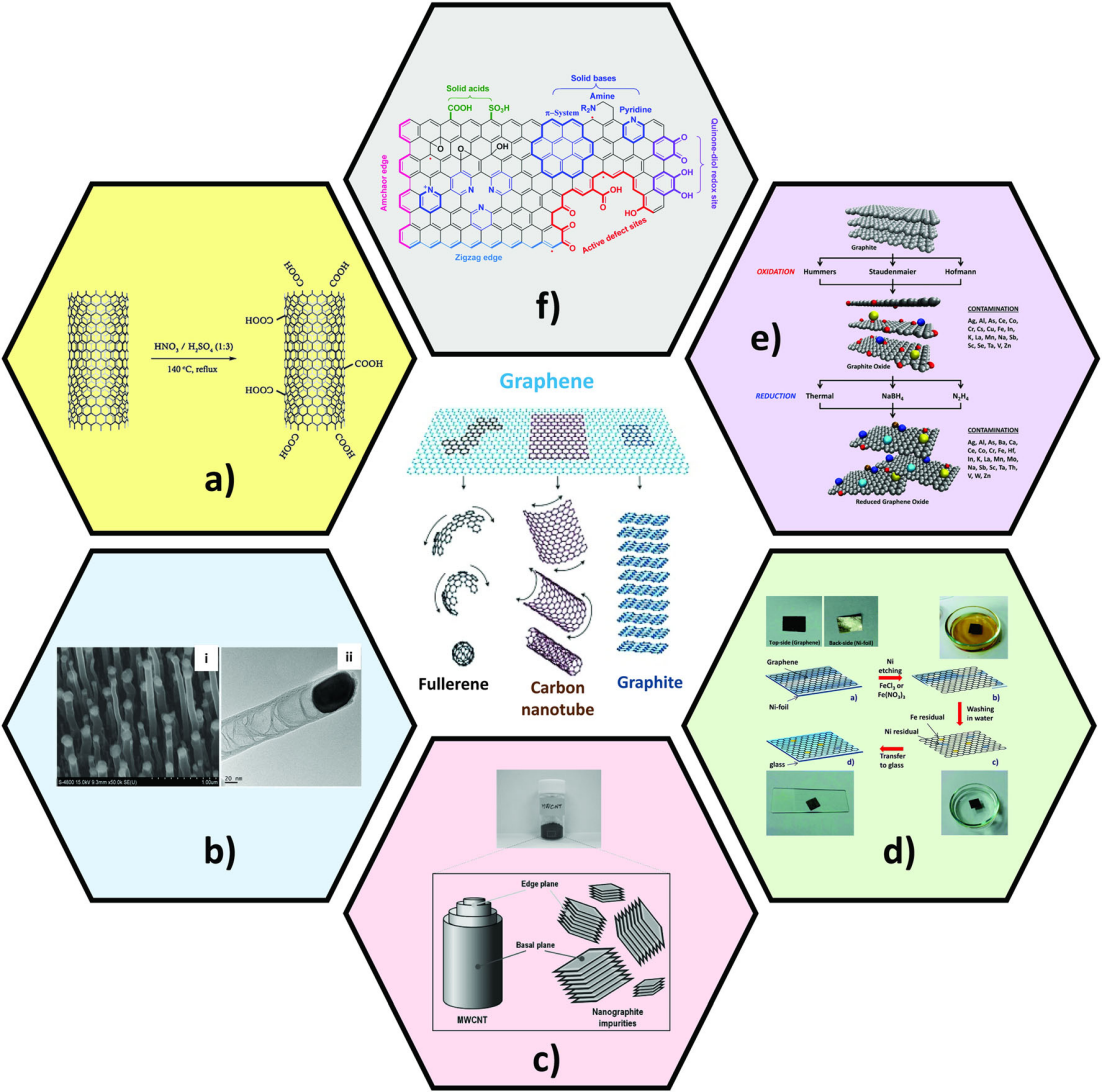
Carbon nanomaterial-based electrocatalysts and their active sites or sources of catalytic activity for non-enzymatic sensors. Middle: graphene and its different structures [11]. **a** Acid-treated CNT and presence of oxygenated groups at the sidewall and tip [12]. **b** (i) SEM and (ii) TEM images of CNTs grown with 20 nm Fe/Ti underlayer [13]. **c** Nanographite impurities present in multi-walled CNT (MWCNT) powder [14]. **d** Introduction of metallic impurities during transferring CVD graphene onto other substrates [15]. **e** Introduction of impurities during common synthesis methods for the preparation of rGO using graphite as a starting material. Gray spheres represent carbon atoms, red spheres represent oxygen atoms, and other colors represent metallic impurities [16]. **f** Active catalytic sites in the graphene domain due to incorporation of heteroatoms [17]. **a** [12] reprinted with permission from Elsevier. Figure 1 in the middle [11] and **c** [14] reprinted with permission from John Wiley & Sons, Inc. **d** [15] reprinted with permission from the Royal Society of Chemistry. **f** reprinted with permission from [17]. Copyright (2013) American Chemical Society

Carbon nanomaterials are considered not only a potential electrocatalyst through their intrinsic functionalities but also an excellent support for anchoring metallic catalysts. Most intrinsic electrocatalytic activities are contributed through oxygen-rich groups and edge-plane-like sites (defects) or metallic and carbonaceous impurities, and heteroatom doping (Fig. 1) [11]. The oxygenated groups are commonly present in carbon nanomaterials and introduced during acid purification/dispersion or synthesis. These oxygenated groups and edge sites can either promote or inhibit heterogenous electron transfer, which is highly dependent on the electrochemical behavior of the analyte [18]. Due to the presence of oxygen-containing groups in graphene sheets, the adsorption through electrostatic means of positively charged organic species, e.g., NAD^+^ and dopamine, can be enhanced [19, 20], thus enhancing electron transfer kinetics and electrocatalytic activity. These groups are also beneficial for anchoring metal-based nanocatalysts. Metallic and carbonaceous impurities are also considered as a main source of electrocatalytic activity in carbon nanomaterials. In particular, metals are involved in the syntheses of carbon nanomaterials. For example, growing carbon nanotubes (CNTs) by chemical vapor deposition (CVD) require metal nanocatalysts which typically remain inside the CNTs after production. The synthesis of reduced graphene oxide (rGO) via the popular Hummers method, for instance, starts with the oxidation of graphite into graphene oxide (GO) and results in the introduction of manganese into rGO. Carbonaceous nanographites and amorphous carbon that are present after carbon nanomaterial syntheses exhibit defects and dangling bonds in a considerably high amount, thus enabling greater electrochemical activity [20]. Carbon nanomaterials doped with heteroatoms have become an attractive catalytic material for non-enzymatic electrochemical sensors [21]. Nitrogen, boron, phosphorus, or sulfur can be doped into the carbon lattice, generating either *p*-type (electron acceptor) or *n*-type (electron donor) doping. Such doping alters electronic properties and chemical reactivity of carbon nanomaterials as the size and electronegativity of heteroatoms are different from those of carbon atoms, leading to attractive features for non-enzymatic electrochemical sensors.

### Electronanocatalysts

This section intends to give aspects on materials that are currently used as well as how they are designed and fabricated to serve as a transducer in non-enzymatic sensors. The challenges of current fabrication strategies will be discussed.

#### Metal-based catalysts

Noble and non-noble metals have long been developed and are popular electrode materials due to their excellent electrocatalytic activity and ease of modification. With respect to the detection of glucose as an example, electrodes modified with a noble metal, e.g., Pt, as the sensing system enable electrocatalytic oxidation under physiological pH, which is beneficial for direct measurement in a biological sample. In contrast, non-noble metals, e.g., Ni, mostly require an alkaline medium to form a OH_ads_ layer, to retain electrocatalytic activity. The alkaline medium is considered a beneficial aspect as it can prevent surface poisoning by halide ions. Yet, unlike biocompatible noble metals, the need for a strong alkaline medium to enable proper catalytic reaction is a major obstacle for in vivo and real-time monitoring, especially in wearable or implantable sensors. In addition, many non-noble metal oxides are toxic to humans and the environment [22–24]. This challenge still impedes the translation of non-noble metal oxide sensor concepts to final devices or systems useable in biomedical applications. Metallic alloys have caught research attention as an alternative option to single metallic components that can facilitate greater overall sensing performance than individual metals. Various metals can be alloyed to obtain completely new attractive features that cannot be achieved with a single component [25, 26].

In order to generate metallic electronanocatalysts for non-enzymatic electrochemical transducers, electrodeposition of respective metallic salts onto electrode supports remains a gold standard procedure nowadays. However, the method suffers from the following drawbacks. First, the technique could lead to poor reproducibility of sensor fabrication. This problem could be potentially caused by the defects on electrode supports that cause electrochemical reduction sites to differ from a surface without defects. In particular, this undesired heterogeneity would be more prominent when rough and porous electrodes are used. Second, poor adhesion stability between electrodeposited catalyst and electrode surfaces may occur, hindering long-term utility, especially under flow conditions. Lastly, the strategy does not lend itself well for mass production. It is crucial to overcome these issues to enable efficient and massive fabrication of non-enzymatic transducers.

#### Carbon nanomaterials and their derivatives

There is a tremendous interest in using carbon nanomaterials owing to their superior mechanical and chemical stability with relatively low material cost. Ease of modification and outstanding electron transfer capabilities also put them into focus for electrochemical sensors, especially when they are combined with non-precious metal oxide nanocatalysts that inherently possess poor electrical conductivity. Carbon nanomaterials comprise a variety of derivatives such as graphene, rGO, graphite, fullerene, heteroatom-doped carbon (nitrogen, boron, chlorine, phosphorus, fluorine, sulfur), nano- or macrostructures such as carbon nanofibers/-tubes, flower-alike, cubes, onions, and various combinations of those [27–29]. In this section, carbon nanomaterials with low dimensions (0D to 2D) will be mainly discussed as they are the explanatory basis for electrocatalytic ability at the more complex dimension.

*Carbon nanotubes* (*CNTs*) consist of graphene sheet/s rolled into cylindrical shape (Fig. 1 middle). As discussed in the previous section, electrocatalytic activity is attributed to oxygen-rich groups present at the edges (tips) or sidewalls of CNTs after acidic treatments (Fig. 1a). However, this is not the case for all analytes, as proven by Gong et al. [18] who thoroughly demonstrated the roles of CNT tips, sidewalls, and oxygen-rich groups for common redox markers, e.g., K_3_[Fe(CN)_6_], nicotinamide adenine dinucleotide (NADH), ascorbic acid (AA), cysteine, and H_2_O_2_. Here, the oxygenated groups at the sidewalls and tips of CNTs facilitated electron transfer for NADH, AA, O_2_, and cysteine, whereas no positive effects were observed for K_3_[Fe(CN)_6_] and H_2_O_2_. It is worth to mention that even though introducing oxygen-functional groups can enhance electron transfer kinetics for some analyte species, the groups can adversely affect electrical conductivity of CNTs as electron delocalization is interrupted by the formed sp^3^-bonded carbon atoms. The CNTs or other carbon nanomaterials with high oxygen to carbon ratios result thus in lower electrical conductivity. Therefore, a balance between electrical conductivity and electrocatalytic ability needs to be met to enable high sensing performance of non-enzymatic sensors based on CNT or other carbon nanomaterials. Synthesis of CNTs is typically conducted by CVD. Here, metal nanoparticles are commonly used as catalysts to grow CNTs from precursor gas. Thus, a great content of metal nanocatalysts still remains inside the CNTs (Fig. 1b), which is considered a major source of electrocatalytic sites in CNTs rather than oxygen-rich groups. An interesting study shown by Jama et al. [30] who have proven that CNTs without iron oxide nanocatalysts did not exhibit electrocatalytic activity for hydrazine whose heterogeneous charge transfer kinetics are sluggish but highly sensitive to the presence of metallic nanocatalyst. Apart from metallic impurities, nanographites are commonly present after the synthesis of CNTs. Nanographites (or carbon quantum dots) consist of multiple graphene layers that are relatively small in their sizes (<10 nm). Thus, nanographites possess a larger number of edge-plane sites per gram of materials than CNTs which greatly contribute to electrocatalytic ability as studied by Ambrosi and Pumera (Fig. 1c) [14]. In particular, the authors have demonstrated improved electron transfer kinetics when the composition of nanographites was increased.

*Graphene and its derivatives* have grown in popularity since their discovery in 2004 [31]. Similar to CNTs, their electrocatalytic activities are attributed to edge sites and defects present in the basal plane. Therefore, pristine graphene grown by CVD is considered a non-preferential material for non-enzymatic electrochemical sensors. However, as shown by Ambrosi and Pumera, the nevertheless observed electrocatalytic effect of CVD graphene mainly resulted from the transferring process in which residual metal catalysts from graphene synthesis and metal-containing etching solution were present (Fig. 1d) [15].

**Fig. 2:**
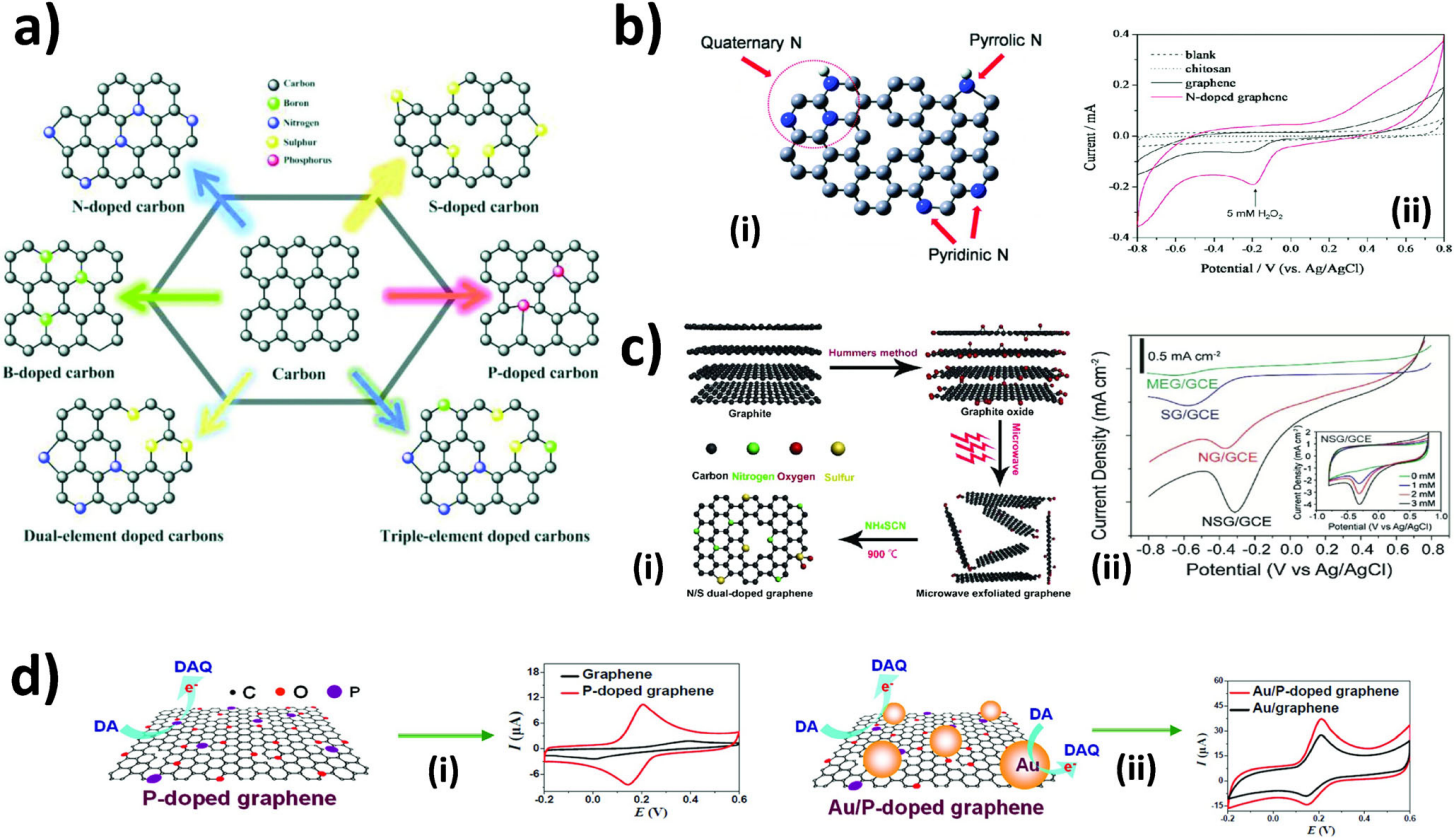
Heteroatom-doped carbon nanomaterials. **a** Structures of various heteroatom-doped carbon [33]. **b** Schematic representation of N-doped graphene. Gray for the carbon atom, blue for the nitrogen atom, and white for the hydrogen atom. A possible defect structure is shown in the middle of the ball-stick model (i), and its electrochemical performance for H_2_O_2_ detection in comparison to conventional electrodes (ii) [34]. **c** Preparation of N and S dual-doped graphene (NSG) (i) and its electrochemical performance for H_2_O_2_ detection in comparison to without N and S (microwave-exfoliated graphene, MEG) and individual atom doping (S-doped graphene, SG, and N-doped graphene, NG) [32]. **d** Electrocatalytic activity of P-doped graphene for dopamine (DA) detection (i), and the improved signal intensity realized by Au/P-doped graphene (ii) [35]. **a** [33] reprinted with permission from John Wiley & Sons, Inc. **b** adapted with permission from [34]. Copyright (2010) American Chemical Society. **c** [32] and **d** [35] adapted with permission from Elsevier

On the contrary, the synthesis methods that produce defects in graphene with compromised electrical conductivities are more favorable. Hummers method is a well-known top-down approach in preparation of graphene oxide (GO). It is based on separating graphene layers from graphite powder through chemical oxidation in a mixture of H_2_SO_4_, NaNO_3_, and potassium permanganate. Further reduction of GO by chemical, thermal, or electrochemical approaches subsequently renders reduced graphene oxide (rGO) which is extensively used in electrochemical sensors nowadays. Here, it should be noted that Mn is the main metallic impurity in rGO prepared via Hummers method and may lead to misinterpretation of electrocatalytic activity of rGO in non-enzymatic electrochemical sensors (Fig. 1e). Apart from graphene with high numbers of oxygen-containing groups, many researchers have focused their interest on heteroatom-doped graphene (Fig. 1f) [21]. Doping graphene with foreign atoms (dopants) that are capable of accepting or donating electrons potentially enhances the performance of electrochemical sensors in general because they promote charge transfer, adsorption, and activation of analytes. A study shown by Xi et al. [32] has demonstrated N and S dual-doped graphene. The authors highlighted the synergistic effect realized from N and S atoms that plays a key role in the activation of carbon atoms of GO structures. Such activation resulted in greater activity of electrocatalytic sites for the decomposition of H_2_O_2_ than that of microwave exfoliated graphene with the individual atom counterparts (Fig. 2c ii).

## Hybrids of nanocatalysts and 3D-carbon nanomaterials

**Fig. 3:**
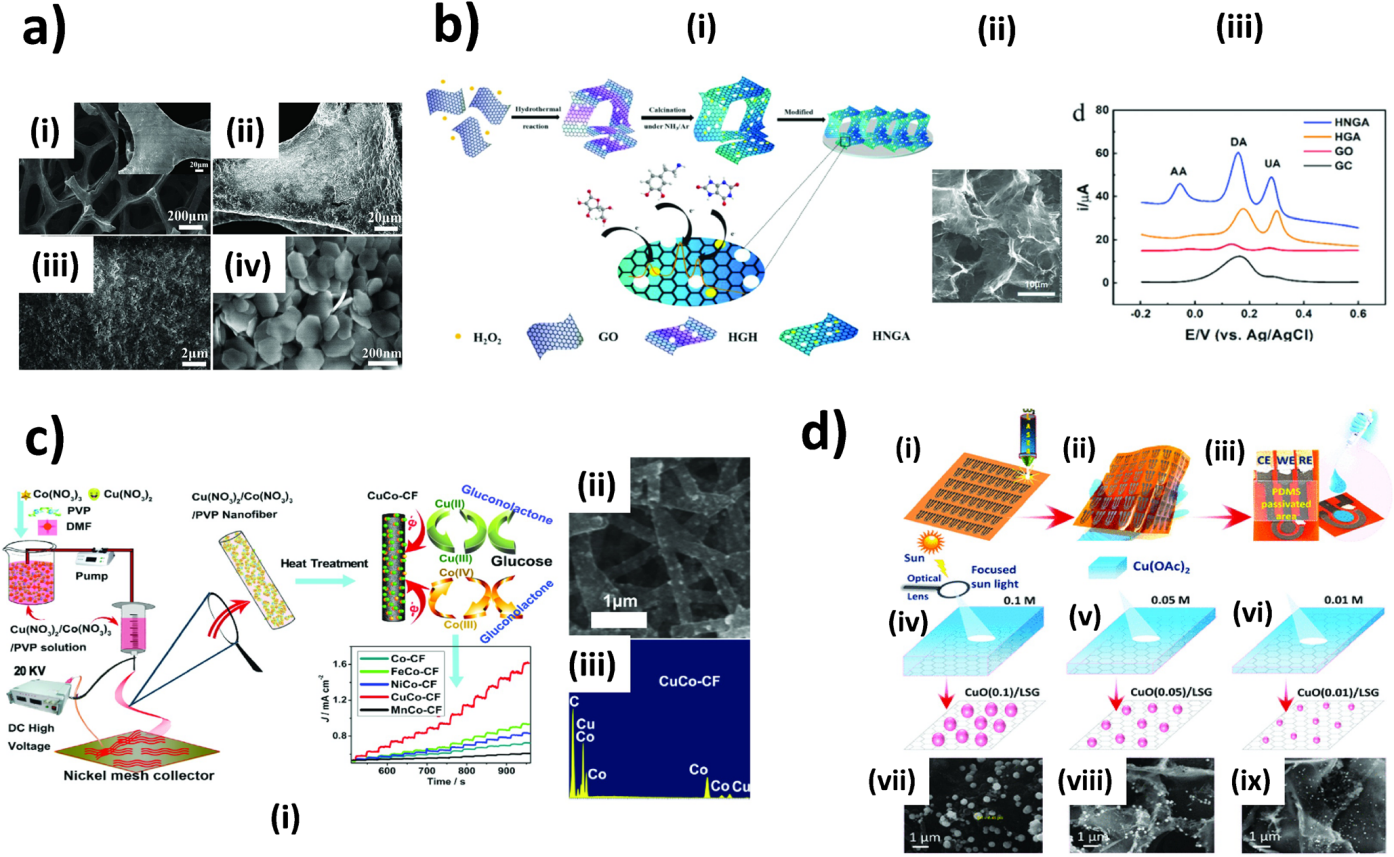
3D carbon nanomaterials–electrocatalyst hybrids for non-enzymatic sensors. **a** SEM image of bare 3D graphene foam (i) where the inset shows the high-magnification SEM image. (ii) and (iii) low- and (iv) high-magnification SEM images of Ni(OH)_2_/3D graphene foam [40]. **b** Hydrothermal preparation of holey nitrogen-doped graphene aerogel (HNGA) (i), SEM image of holey graphene aerogel (HGA) (ii), and electrochemical performance of the electrodes for simultaneous detection of AA, DA, and UA (iii) [41]. **c** Fabrication of bimetallic CoCu-carbon fiber (CuCo-CF) via electrospinning and thermal carbonization, and electrochemical performance of various bimetallic electrocatalysts (i), morphological structure of CuCo-CF after thermal carbonization (ii), and its energy dispersive X-ray (EDS) spectra (iii) [42]. **d** Patterning laser-scribed graphene (LSG) electrodes followed by drop-casting of Cu salt precursor solution (i–iii), reduction of Cu precursor via irradiation by focused sunlight (iv–vi), and SEM images of LSG-CuO nanoparticle hybrids prepared from different concentrations of Cu precursors (vii–ix) [39]. **a** [40] reprinted with permission from the Royal Society of Chemistry. **b** [41] and **c** [42] adapted with permission from Elsevier. **d** reprinted with permission from [39]. Copyright (2020) American Chemical Society

Metallic nanocatalysts and carbon nanomaterials possess distinct electrocatalytic sites and mechanisms as described in the previous section. Noble metal-based nanocatalysts exhibit great electrocatalytic activity, especially under physiological pH, but high propensity to surface poisoning and low abundance with relatively high cost make them less attractive. Non-precious metals in the form of oxides have long been developed as an alternative choice. However, poor electrical conductivity of many metal oxides makes supports with efficient electron transfer necessary. Carbon nanomaterials are thus extensively employed to serve this task [36]. 3D-porous carbon nanomaterials have been proposed as great candidates for anchoring electrocatalysts alternative to using 1D- or 2D-nanomaterials, especially when the electrodes are integrated into miniaturized analytical devices where numbers of analytes are being limited. The network of carbon nanomaterials can potentially promote efficient diffusion inside miniaturized systems and thus enhance detection sensitivity. Graphene foam [37], carbon nanofibers [38], and laser-induced graphene (LIG) [39] that were decorated with electronanocatalysts have proven their excellent analytical performance as non-enzymatic electrochemical transducers (Fig. 3). Alternatively, the possibility to introduce heteroatoms during or after the synthesis of such 3D-carbon nanomaterials is likely to advance the field significantly. In this section, we therefore want to highlight the current state of the art in preparation of these hybrids as free-standing electrodes for non-enzymatic sensors and their pros and cons.

### Graphene foam

In general, the preparation of graphene composites suffers from agglomeration of graphene sheets due to strong π–π interaction, thus diminishing available active surface area for functionalization with nanocatalysts and electrocatalytic reaction. 3D-graphene foam has been developed to overcome this issue. It can be synthesized with various methods as described by Baig and Saleh [43]. These methods include CVD, hydrothermal process, lithography-based techniques, support-assisted synthesis and chemical deposition, and direct electrochemical deposition. CVD is more frequently applied for 3D-graphene foam generation than other techniques. Here, a 3D-porous metallic catalyst, e.g., Ni foam, is employed as a scaffold to grow graphene sheets by CVD instead of a flat metallic surface as used for 2D-graphene synthesis [43]. The metallic scaffold is later etched away by a strong oxidant, e.g., hot HCl (or FeCl_3_). However, prior to etching, it is necessary to coat the as-fabricated graphene foam with a thin layer of polymer, e.g., poly(methyl methacrylate) (PMMA), to prevent the structure from collapsing during the etching process. Typically, the pore diameters of a graphene foam network fall into tens to hundreds of micrometers, depending on the pore sizes of the metallic scaffold (Fig. 3a). In order to decorate graphene foam with metallic nanocatalysts, various strategies can be employed, the most popular of which is in situ hydrothermal synthesis [44]. In 2012, Dong et al. [37] grew cobaltosic oxide (Co_3_O_4_) nanowires (a few hundreds of nanometers in diameter) on graphene foam via this technique. Here, the as-synthesized graphene foam fixed on a glass slide was exposed to a solution containing CoCl_2_·6 H_2_O and urea in an autoclave at high temperatures for several hours. The as-prepared hybrid demonstrated a remarkable sensitivity for glucose with a LOD as low as 25 nM. It should be noted that for this approach the growing conditions, i.e., precursor concentration, reaction temperature, and time, greatly control the overall morphology of nanocatalyst that can subsequently affect analytical performance [45]. Therefore, forming various structures of nanocatalyst on graphene foam surfaces, e.g., hexagonal Ni(OH)_2_ nanosheets [40], and Cu(OH)_2_ flower-like structures consisted of nanorods [46], would be possible. Simple adsorption of pre-synthesized nanoparticles on graphene foam has also been proposed as an alternative to hydrothermal synthesis [47]. In this work, the authors have proven that the large available surface area of graphene foam increased the number of binding sites of PtRu bimetallic nanocatalysts, and thus subsequently promoted efficient electron transfer and mass transport of analyte to the nanocatalysts. As expected, the sensors exhibited an excellent analytical sensitivity for H_2_O_2_ sensing with a LOD as low as 40 nM which was an order of magnitude lower than with 2D graphene. A recent study by Usman et al. [48] demonstrated a more sophisticated version of 3D-carbon foam decorated with silver nanoparticles that enabled the detection of glucose with outstanding sensitivity and LOD (6 nM). Here, carbon nanocoils were grown on a porous nickel template by CVD prior to forming silver nanoparticles on the carbon nanocoils via electrodeposition under continuous stirring. However, it should be noted that it was necessary to render the electrode surface hydrophilic before electrodeposition. In this case, they immersed the electrode in a solution of the detergent cetyltrimethylammonium bromide (CTAB) under continuous stirring for 12 h to enhance the hydrophilicity of the electrode surface. This is also of importance to other porous carbon electrodes which are commonly hydrophobic and composed of micro-/nanostructures, thus hindering the accessibility of aqueous reactant (with high surface tension) to the reaction sites available on the electrodes. Graphene foam doped with nitrogen or other heteroatoms can be synthesized by CVD when a nitrogen precursor, e.g., ethylenediamine, is introduced during the growth of graphene on the metallic porous template [49].

Hydrothermal reduction of GO into 3D-graphene or rGO hydrogel is an alternative simple approach that does not require a template [50]. The reaction is typically carried out at a high temperature for several hours to thermally reduce GO sheets which subsequently form a 3D random stack of flexible graphene sheets through hydrophobic and π − π interactions (Fig. 3b). The 3D-graphene hydrogels typically possess pore sizes in the range of sub-micrometers to several micrometers. However, the GO concentration critically governs the successful formation of a 3D network. At low GO concentration, cross-linking of graphene networks is difficult, and aggregates form and precipitate as powders. Addition of a catalyst precursor, e.g., metal salt (metal nanoparticle source) [51] and dopamine (nitrogen dopant) [52], during the hydrothermal process enables the incorporation of catalytic activity to the 3D-graphene hydrogels in a single step.

Even though graphene foam decorated with electronanocatalysts has proven its excellent sensitivity in nM ranges, the strategy is still suffering from the following drawbacks: (1) graphene foam generation involves high production cost using sophisticated instruments under strictly controlled conditions, and (2) difficulties in patterning electrode as well as integration into miniaturized analytical systems [53, 54].

### Carbon nanofibers

Carbon nanofibers (CNFs) consist of stacked graphene sheets of various orientations where edge sites are exposed to the exterior of the structure, featuring more active sites than CNTs [55]. Their self-forming 3D-interconnected porous structure makes CNFs highly attractive as electrochemical transducers. Currently, there are two widely used methods for CNF fabrication which include CVD and electrospinning technique combined with subsequent carbonization. While the former technique offers CNFs with high quality, the latter strategy is more economic due to the relatively lower cost of instrumentation. Therefore, electrospinning combined with a carbonization process is more attractive for mass-fabricating non-enzymatic electrochemical sensors. Electrospinning is a technique used to generate a nanofiber precursor with diameters in the range of a few hundreds of nanometers. The setup is simple and includes (1) a high voltage supply, (2) a continuous feeding system, and (3) a collector. In principle, a high voltage is applied to a polymer solution that is continuously fed into the system. Increasing the voltage until the repulsive forces of the charged polymer overcome the surface tension of the formed droplet results in a polymer jet ejecting towards the collector (Fig. 3c). The process is not only efficiently controllable but also highly facile and well-suited for mass production scales. Thermal treatment of the as-spun nanofiber precursor is subsequently performed to render CNFs [56].

In order to generate non-enzymatic transducers from CNFs, ex situ or in situ generation of nanocatalysts can be employed. Electrochemical reduction of metal salt onto electrospun CNFs has been commonly employed in an ex situ-based method [57]. However, as discussed for graphene foam, the accessibility of deposited catalyst on the entire surface area needs to be ensured to achieve good reproducibility between as-fabricated sensors. The more convenient and popular technique relies on an in situ–based approach where the metal salt precursor is co-electrospun with the polymer solution (Fig. 3c i). Upon carbonization, the metal salt is reduced and subsequently transformed into nanocatalyst particles [58]. Previous studies of in situ functionalized nanofibers revealed excellent electrocatalytic activity towards H_2_O_2_ reduction and NADH oxidation [58]. Similarly, as shown by Liu et al. [59], Ni-loaded CNFs exhibited remarkable performance for glucose sensing. This strategy has the following significant merits. First, increasing the ionic conductivity of a spinning solution due to the addition of metal salt typically promotes uniformity of fiber structures (beaded-free fibers). Second, good dispersion of metal salt precursors along the nanofibers is anticipated as a charge from the applied voltage should assist the even distribution of the ionic salt. This may not be the case when preparing film as a substrate where the evaporation behavior critically determines the homogeneity of dispersed ions. Under normal conditions, the unfavorable coffee-ring effect can occur. Third, the nanocatalysts could be firmly embedded inside CNFs, strengthening their mechanical stability in CNF matrix, especially when electrochemical measurements require stirred or flow conditions. The thermal reduction typically creates nanocatalyst particles in the size range of approximately tens to hundreds of nanometers. An interesting study shown by Zhu et al. [60] demonstrated an unexpected behavior of Au nanoparticles (AuNPs) embedded in the interior of electrospun polyacrylonitrile nanofibers, which can migrate to the external surfaces of the CNFs during the thermal carbonization process. Here, heating rate and carbonization temperature play a crucial role in the migration behavior. Slow heating rates and high carbonization temperature were favorable for the formation of small sizes of AuNPs (less than 10 nm) and high density of AuNPs present on the surface, respectively. The smaller size and higher density of exposed nanocatalysts synergistically boosted the electrocatalytic activity of the materials as proven by the application for non-enzymatic detection of H_2_O_2_.

CNFs can be doped with heteroatoms via two methods, including (1) carbonization of heteroatom-rich nanofiber precursors, and (2) carbonization of nanofiber precursors under heteroatom-containing atmosphere. Urea and conjugated polymers, e.g., polyaniline (PANI) and polypyrrole (PPY), can be added to a polymer solution to increase the N content in the nanofiber precursor [61]. Using molecular nitrogen or NH_3_ as a gas carrier during thermal carbonization yields N-doped CNFs [62, 63]. The heteroatom-doped CNFs for non-enzymatic sensors have by far been less explored than other forms of graphene-based materials [21]. Considering the versatility of electrospinning to generate heteroatom-rich nanofiber precursors, various kinds of metal-free CNFs with catalytic ability could be generated which are particularly of interest to biomedical applications.

Despite the remarkable analytical performance of metal nanocatalysts- or heteroatom-doped CNFs in non-enzymatic electrochemical sensors, most of the previous studies still rely on GCE or carbon paste electrode as a support for the CNFs to elucidate the superior electrochemical behavior of the catalyst in comparison to conventional materials, i.e., without modifications [38, 64]. Intrinsic advantages from 3D-porous structure have not been actually realized as the beneficial structures are destroyed by the preparation process, e.g., through sonication or grinding prior to transferring the suspension or paste onto the base electrode. Liu et al. [65] demonstrated a strategy to incorporate a mat of free-standing electrospun N-doped CNFs onto GCE by simply cutting the nanofiber mat and adhering to the electrode surface using Nafion as a binder. Yet, their method may be not suitable for mass production scale.

### Laser-induced graphene

Laser-induced carbonization is an emerging technology aiming to fabricate carbon nanomaterial electrodes from potential precursors in a facile and massive manner. For laser writing, different types of lasers, either pulsed or continuous wave such as the most popular CO_2_ laser (typically 10.6 μm wavelength), can be employed for this purpose [66]. The generation of conductive graphene from a commercially available non-conductive flexible substrate with this technique was first introduced by the group of Tour [67]. Here, graphene electrodes with high porosity can be massively and simply fabricated with high affordability and readily available equipment and precursor materials. This is in contrast to traditional strategies, e.g., thermal carbonization and CVD. Therefore, it is obvious that laser-induced carbonization, which additionally offers electrode design flexibility and device integration ability, is becoming a promising candidate for constructing non-enzymatic electrodes in particular for miniaturized analytical systems. Various kinds of substrates, e.g., wood [68], cloth, food, or paper [69, 70], can be converted into highly conductive graphene-based electrodes which broadens the range of potential applications. Most intensively employed are polyimide sheets (PI, DuPont brand name: Kapton) which act as a precursor for laser-induced graphene (LIG) electrodes (also sometimes wrongly referred to as laser-scribed graphene, LSG).

Commercial laser systems offer the advantage of switchable lenses for a respective desired application. Their architecture results in varying conditions of the focus namely focal length, spot size, and depth of focus. In the focal point, energy and heat density are the highest and decrease equally with increasing or decreasing distance according to the Gaussian beam characteristics [71]. With respect to this, it must also be kept in mind that the thicker a sample the more likely it to have morphological differences through the whole specimen thickness after carbonization. The 2.0″ lens, which is often the standard lens system for CO_2_ devices, can be used for carbonizing or cutting several materials. Due to the rather high tolerance (depth of focus) of 2.54 mm, it is easy to find the correct focus even for materials that do not possess an evenly flat surface. However, this aspect and the average focal point of 127 μm make the lens less suitable for scribing/cutting fine structures. In this case, a high-power density focusing optics (HPDFO) should be employed. As stated by its name, the energy input is concentrated on a very small focus spot (25 μm) allowing very high resolution. This comes also with the drawback of high divergence/low focal range tolerance. In addition, the increased energy per illuminated spot can result in distortion of sensitive materials (e.g., polymer foils). The energy input can be fine-tuned by the dots-per-inch (DPI) setting in the laser software which though affects the duration of the lasing process.

Further software settings of the CO_2_ laser have a direct impact on the generated carbon structure and quality. Focusing on laser-induced graphene, it is of utmost importance to optimize parameters of the system namely lasing power and speed to obtain carbon with high quality. Generally, increasing the lasing power comes with the need of increasing speed as well and longer lasing exposure times should be ensembled with lower power [72]. Hereby, the morphological features, e.g., flake/pore size, layering or gap width, usually deviate from different possible parameter combinations and the choice depends on the preferred application and the energy absorption ability of the material to be carbonized. The energy input by the laser and carbonization respectively can also be tailored by changing the distance of the material surface relatively to the laser focal point. In a later chapter, we discuss the carbonization of electrospun nanofibers with inherent metal complexes, which assist in distributing the laser input energy evenly resulting in high integrity of the carbon nanofiber morphology.

The atmosphere in which the lasing process takes place also has a strong effect on the obtained features. Differences in morphology as well as the introduction of functional surface groups and defects by scribing in ambient conditions compared to controlled gas atmospheres such as O_2_, Ar, and H_2_ lead to significant changes in wettability and electrochemical properties [73]. Lastly, our group found that the scribing direction affects to electrochemical performance of carbonized electrodes [72]. Common CO_2_ laser systems are scribing in one direction, e.g., from left to right, before returning to the starting point and continuing with an adjacent line underneath the one carbonized previously. Therefore, for the carbonization of patterns with differences in *x*- and *y*-length, the number of carbonization lines differs when scribing along the *x*-axis compared to the *y*-axis. This comes with morphological differences such as smaller vs. larger structures (surface area) or pores (-size) that again may further influence electrochemical behavior. Concluding, when developing a new laser carbonization–based composite, the influence of many parameters needs to be studied first and fully understood to customize towards desired material features.

The fabrication of functional materials by laser writing technology has moved into focus due to the simple generation of diverse nanocatalyst-carbon hybrids. LIG modified with various metal nanoparticles, copper [74] or platinum [75], through electrodeposition has proven its excellent electrochemical sensing abilities. However, the mechanical and chemical stability of metal nanoparticles deposited on LIG surfaces is rather low, which may hamper the use in long-term monitoring or under flow and stirred conditions. It was demonstrated, also for the first time by Tours’ research group, that metal oxides can be embedded into graphene by doping the PI precursor (polyamic acid or PAA) with a metal salt and subsequently carbonize [76]. The obtained LIG contained nanocrystals and showed remarkable electrocatalytic activity for the oxygen reduction reaction (ORR). This work has triggered several other research groups to start fabricating hybrids of laser-induced graphene with metal composites especially in energy storage-related fields. However, considering the applications in non-enzymatic electrochemical sensors, only few studies have been proposed so far.

Many of the suggested fabrication techniques for energy storage-related electrodes are in fact well-applicable to non-enzymatic electrochemical sensors. The most popular strategy is to create a polymer film consistinged of metal nanocatalyst precursor prior to undergoing laser carbonization [77, 78]. Great adhesion stability between the nanocatalysts and carbon matrices could be anticipated with this strategy. Alternatively, the functional materials can be applied onto the surface of the PI sheet and then exposed to the laser in order to form the functional carbon hybrids [79, 80]. Here, the functional nanocatalysts are exposed to the exterior of the transducer materials which is beneficial for achieving greater analytical performance as compared to the former technique. However, adhesion stability may remain a crucial question. Mixing the metal precursor into a polymer matrix, e.g., chitosan, and strengthening its binding to the PI sheet through hydrophilic reagent as proposed by You et al. [81] could reasonably overcome this problem. Apart from single metal species, future concepts could involve the preparation of metal alloy-carbon hybrids with multiple functions directly by laser. Similar to the laser surface alloying technique, where commonly a metal powder is distributed on a surface of another metal (e.g., chromium on copper [82] or aluminum on titanium [83]) and alloyed afterwards by laser power, different metal powders could be alloyed and united with carbon matrix at the same time.

Heteroatom-doped graphene can also be generated via the laser writing technique as an alternative to common costly methods such as (hydro)thermal annealing or plasma synthesis. As an example, a simple route of doping LIG with boron (B) atoms is shown by Peng et al. [84] who mixed poly(amic acid) with boric acid. After thermal imidization, the B-doped PI film was laser-carbonized. The resulting B-LIG possessed multiple times enhanced areal capacitance as well as energy density which make it a promising candidate for microsupercapacitor development [84]. In a similar way, N-doped LIG was achieved via urea/PAA precursor. An N-doping level as high as 13% could be obtained under laser writing in an ambient atmosphere. However, the doping level could be even more increased when the laser writing was performed under N_2_ atmosphere [85]. Kim et al. [86] presented a different approach of preparing N-doped LIG which is by laser carbonization of a polyimide layer that was deposited on LIG that has been carbonized from an initial polyimide layer before. The authors propose that the significantly increased nitrogen content of densified LIG results from in situ doping deriving from the second polyimide layer [86]. An effective manner to synthesize sulfur-doped graphene is to use sulfonated poly(ether ether ketone) as LIG precursor instead of polyimide as demonstrated by the studies of Lamberti et al. [87].

### Laser-induced carbon nanofibers

**Fig. 4:**
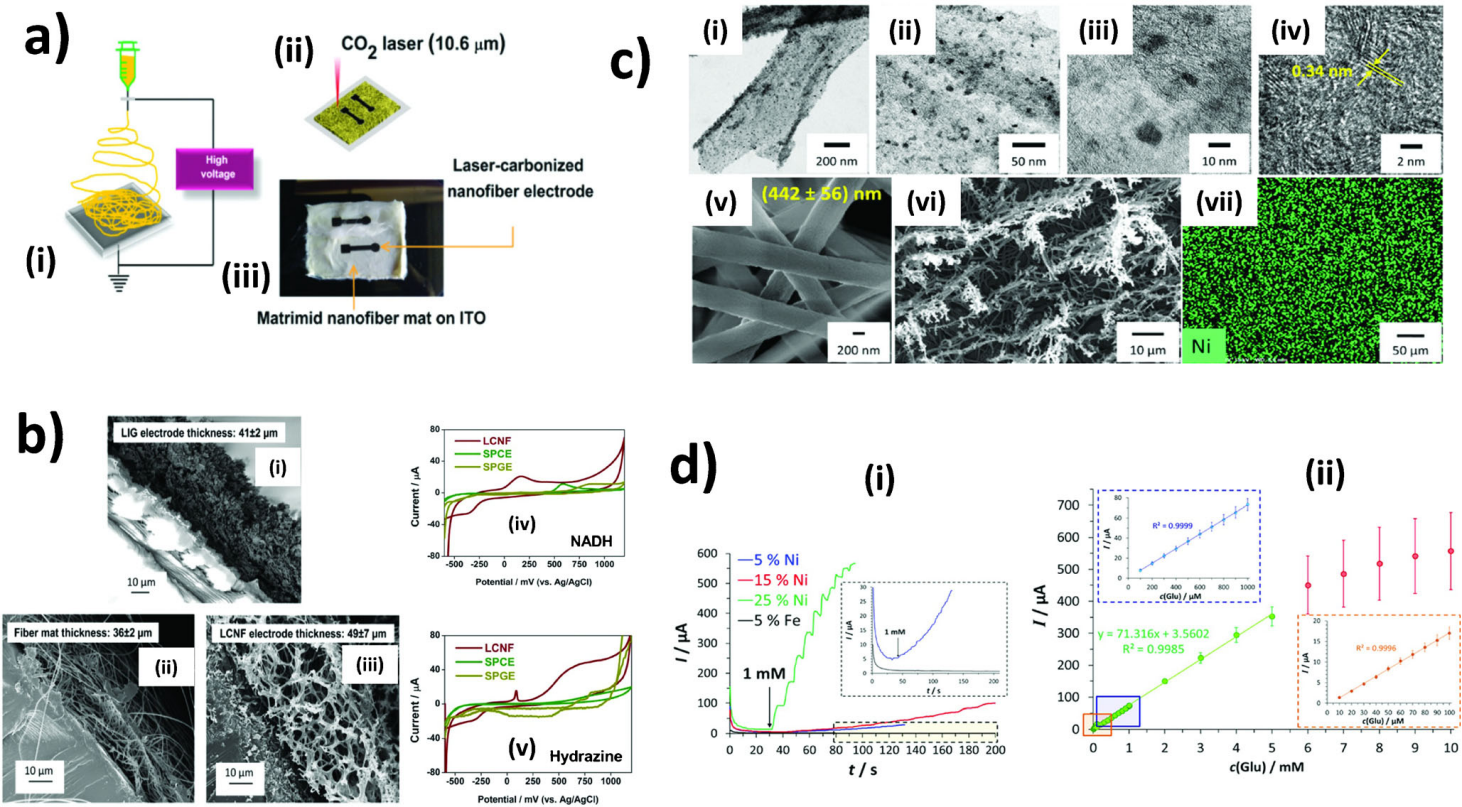
Laser-induced non-enzymatic carbon nanofiber hybrids. **a** Fabrication of nanofibers precursors via electrospinning and carbonization by irradiation using CO_2_ laser (i and ii), and a photograph of a laser-induced carbon nanofiber (LCNF) electrode (iii) [88]. **b** SEM images show the side view of LIG, electrospun nanofibers before (ii) and after laser carbonization (iii), electrocatalytic activity of LCNFs containing Fe in comparison to screen-printed carbon (SPCE) and gold (SPGE) electrodes (iv and v) [88]. **c** TEM images from low- to high-magnification display the presence of Ni nanoparticles in LCNFs after laser writing (i–iii), stacked graphene sheets present within LCNFs (iv), morphological structure of as-spun nanofibers containinh Ni before (v) and after (vi), and distribution of Ni on LCNF electrode studied by EDS (viii) [89]. **d** Amperograms of glucose at various LCNF electrodes (i), and calibration plot of glucose obtained from LCNFs with 25% Ni (ii) [89]. **a** and **b** [88] adapted with permission from the Royal Society of Chemistry. **c** adapted and **d** reprinted with permission from [89]. Copyright (2020) American Chemical Society

As mentioned in the previous section, conventional fabrication strategies for nanocatalysts-CNFs hybrids suffer from some drawbacks such as tedious electrode preparation, inflexibility of electrode design, loss of beneficial features after processing, and incompatibility with large-scale production. Combining electrospinning and laser-induced carbonization/functionalization (Fig. 4a) is a promising strategy to overcome such challenges. Our research group has recently demonstrated the feasibility of the technique in generating high-performance non-enzymatic electrochemical transducers under ambient conditions [88, 89]. We initially investigated fabrication parameters that critically govern the morphology and electrochemical properties of the laser-induced carbon nanofibers (LCNFs) [88]. Here, we found that the electrochemical behaviors of the as-prepared LCNFs are strongly determined by their morphological structures. Unlike the PI sheet, density and thickness of electrospun nanofiber mats played a significant role in obtaining LCNFs with high integrity. Therefore, electrospinning parameters such as spinning time, tip-to-collector distance, and collecting area have to be carefully considered. In addition, the intrinsic heat condition property of the as-spun fibers is of utmost importance to maintain the structural integrity and quality of LCNFs. The addition of metal salt into the spinning solution promotes efficient heat dissipation along the nanofibers during the laser carbonization process (Fig. 4c). Furthermore, the lasing parameters, especially the laser beam size, laser speed, laser power, and lasing strategy, play a crucial role in determining carbon quality which hence further reflects to electroanalytical performance.

In addition to the advantages realized from electrospinning and laser-induced carbonization, the resultant LCNF electrodes exhibit high porosity that cannot be obtained from the use of PI film (Fig. 4b i–iii). Therefore, LCNFs are more suitable than PI film for integration into microfluidic analytical systems as the LCNFs allow for greater percolation of fluids. The estimated pore sizes of LCNF electrodes are ca. two orders of magnitude smaller than the typical reports for graphene foam prepared by CVD [90]. This can enhance efficient interaction between the analyte and the functional interface. Furthermore, unlike thermal treated CNFs that commonly possess intact fibrous structure due to heat stabilization step, the imperfect fibrous structures of LCNFs, i.e., partial broken LCNFs especially at the top part, may contribute to a greater number of edge defects which can facilitate electrocatalytic reaction (Fig. 4b iv and v, Fig. 4d). Lastly, it has been proven in our work that functional nanocatalysts, Ni in this case, can be generated by CO_2_ laser and are distributed evenly along the LCNFs (Fig. 4c). The laser-generated nanocatalysts adhered more firmly to the LCNFs that electrodeposited nanocatalysts when applied to hydrodynamical forces (e.g., shaking in buffer solution for 5 h). Undoubtedly, the Ni-LCNF electrodes exhibited remarkable electrocatalytic activity for the oxidation of glucose. The strategy is practical for generating CNFs doped with not only metal or metal oxide nanocatalysts but also with heteroatom dopants.

## Advances in laser-induced (functional) carbon nanomaterials in sensing applications

In this section, we aim to point out existing sensing platforms based on laser-induced carbon nanomaterials which can further guide readers to imagine how they can be integrated or adapted in non-enzymatic sensor applications. In addition, various alternative approaches for creating the hybrids that are not mentioned in the previous sections are also included here.

**Fig. 5:**
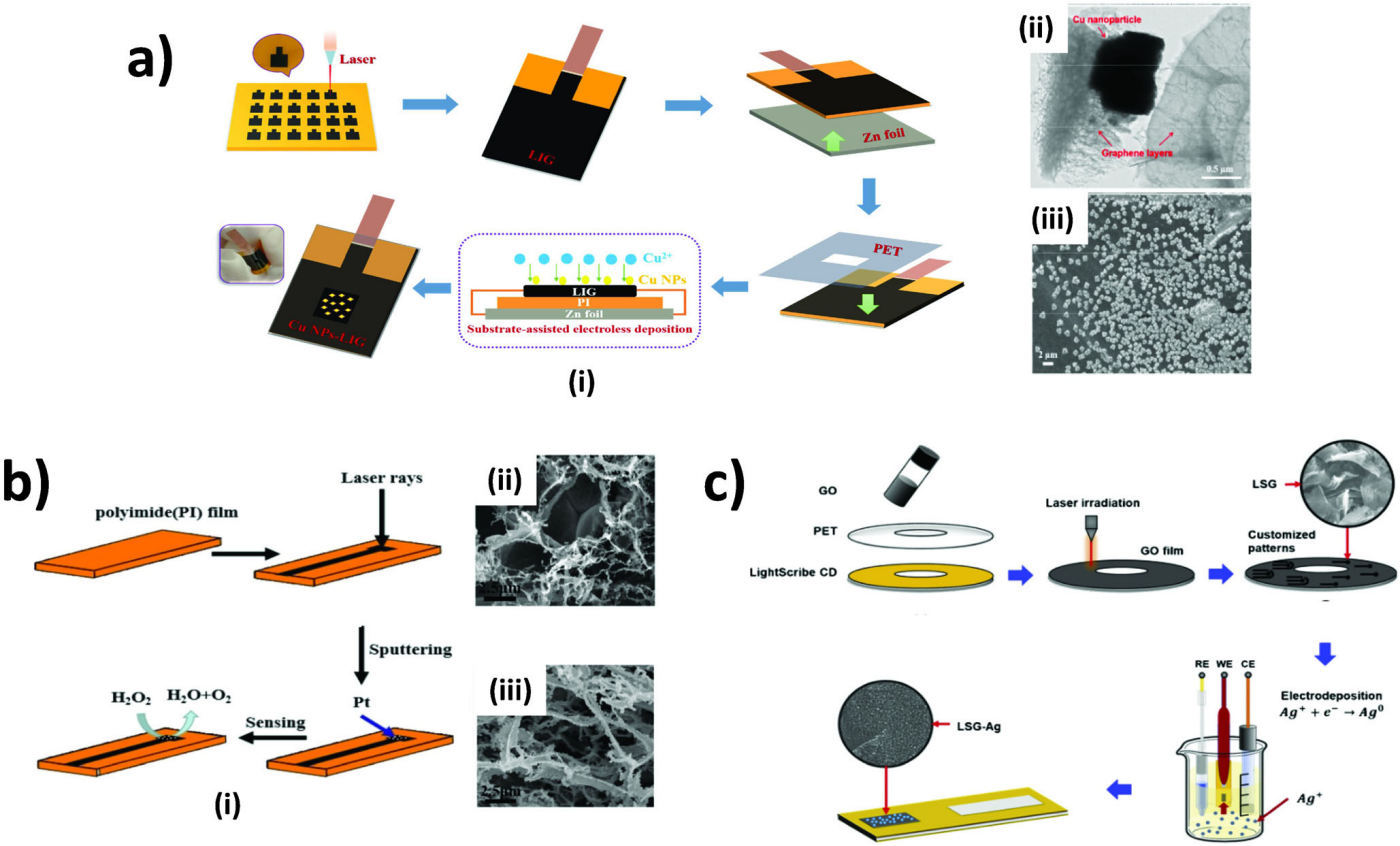
Various approaches for fabrication of laser-induced carbon nanomaterials and metal hybrids in sensing applications. **a** Modification of LIG with Cu NPs through substrate-assisted electroless deposition (SAED) (i), TEM images of cubic Cu NP decorated LIG with high (ii) and low (iii) magnifications [91]. **b** Modification of LIG via sputtering (i), and SEM images of LIG before (ii) and after (iii) Pt sputtering [92]. **c** The schematic illustration of electrodeposition of Ag onto LSG electrodes [93]. **a** [91] adapted and **c** [93] reprinted with permission from Elsevier. **b** [92] adapted with permission from John Wiley & Sons, Inc

As the mechanical stability of LIG is sufficient, it has already been developed towards sensing of multiple analytes in aqueous media. In the past few years, LIG electrodes have been fabricated and later modified with metal nanocatalysts via various techniques as described in the following examples. Zhang et al. [91] decorated LIG, which was generated on a PI sheet, with copper nanoparticles (Cu NPs) by substrate-assisted electroless deposition (SAED). They connected LIG to zinc foil and immersed it into copper solution as illustrated in Fig. 5a i. Due to the potential difference between the zinc and the copper ions, copper nanoparticles are deposited on the LIG surface. The SAED enabled the formation of cubic Cu NPs with a relatively large size (the length approx. 800 nm) (Fig. 5a ii and iii). They were able to amperometrically quantify glucose in the range of 1 μM to 6 mM with a LOD of 0.39 μM. Also, they could detect glucose from a human blood serum sample that was injected into a NaOH matrix without significant signal interference [91]. Sputtering and electrodeposition are also common methods to modify laser-induced carbon electrodes. For example, Zhang et al. [92] generated LIG electrodes on a PI sheet and Pt was later sputtered on the electrodes (Fig. 5b). The electrodes were employed for H_2_O_2_ sensors with considerably low LOD (sub-micromolar range). Besides, the authors have demonstrated the utility of the sensors for spiked H_2_O_2_ in culture medium for mammalian cells. However, the spiked concentrations of H_2_O_2_ were investigated in the range of hundreds of micromolar, implying that further improvement in detection sensitivity has to be performed to enable the monitoring of H_2_O_2_ release from the cells. In addition to sputtering, electrodepositing was employed for generating Ag nanoparticles onto graphene electrodes which were prepared from GO film coated onto a plastic film (Fig. 5c) [93]. The electrodes enabled H_2_O_2_ detection with a wide linear range and low LOD as well as good recoveries when H_2_O_2_ was spiked in skimmed and whole milk. Furthermore, the authors demonstrated that continuous bending of the electrodes for several times only caused a slight loss of current signal (~13%) which makes them highly suitable as wearable sensors. Overall, although the proposed LIG decorated with metal catalysts exhibited favorable analytical performance in the proof-of-principle, the fabrication methods yet involve tedious process and may not lend themselves well for mass production. In addition, functionalization of LIG through post-modifications may result in poor adhesion stability between the metal nanocatalysts and LIG surface, suggesting that generating PI film with functional precursors is more favorable.

**Fig. 6:**
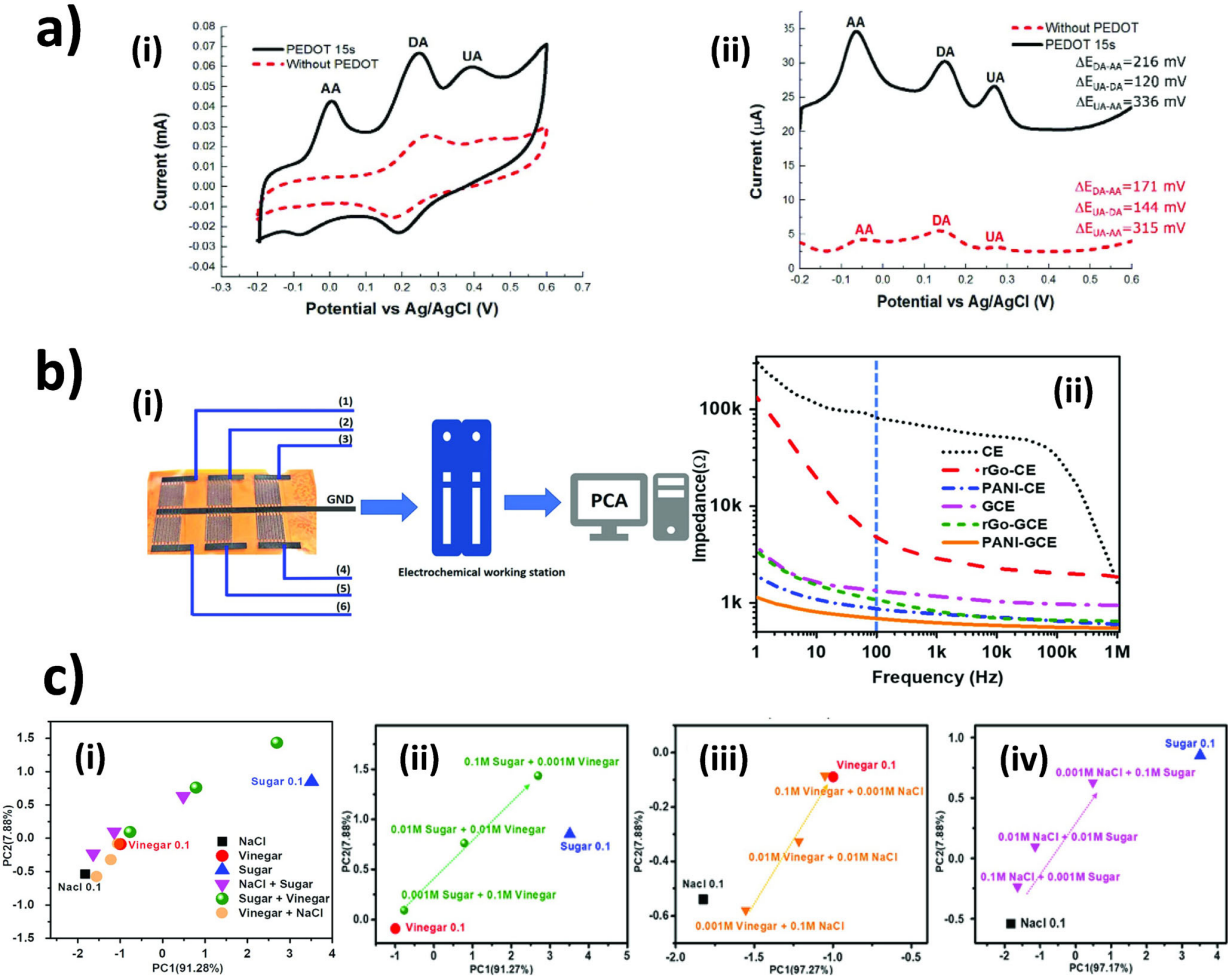
Multi-analyte detection realized by LIG electrodes. **a** Simultaneous detection of AA, DA, and UA by DPV (i) and CV (ii) on LIG electrodes [94]. **b** Sensing system setup (i) and alternating current impedance response for distilled water (ii). **c** PCA plot for impedance responses of (i) mixed solutions and enlarged views for the responses of the (ii) vinegar and sugar mixture, (iii) NaCl and vinegar mixture, and (iv) NaCl and sugar mixture [95]. **a** [94] reprinted with permission from Elsevier. **b** reprinted and **c** adapted with permission from [95]. Copyright (2018) American Chemical Society

Multi-analyte detection realized by LIG electrodes using voltammetric-based techniques has been proposed (Fig. 6). However, employing bare LIG electrodes may not result in well-resolved peaks which requires additional surface modifications as reported by the following examples. Xu et al. [94] demonstrated the feasibility of simultaneous detection of ascorbic acid (AA), dopamine (DA), and uric acid (UA) on unmodified LIG electrodes using differential pulse voltammetry (DPV). However, when modified the electrodes with poly(3,4-ethylenedioxythiophene) (PEDOT) the anodic peaks of AA, DA, and UA were considered higher and better resolved than unmodified LIG electrodes (Fig. 6a). In particular, the PEDOT modified LIG electrodes enabled cyclic voltammetry (CV) to resolve anodic peaks of the analytes. Nayak et al. [75] have demonstrated that modification of LIG with Pt NPs not only promotes electron transfer kinetic of LIG but also improves electrocatalytic performance in simultaneous detection of AA, DA, and UA as can be seen from larger peak separation in comparison to pristine LIG electrodes. A more advanced solution for the detection of many analytes in an aqueous mixture was presented by Yu et al. [95] who fabricated a LIG sensor array based on the electronic tongue principle (Fig. 6b). Their systems consist of six units that are each functionalized with different composites such as gold, rGO, and polyaniline (PANI) (Fig. 6b). By principle component analysis (PCA) of impedance data, their sensor was able to distinguish between sweet (sugar), salty (NaCl), and sour (vinegar) at thresholds lower than possible for a human tongue (Fig. 6c).

**Fig. 7:**
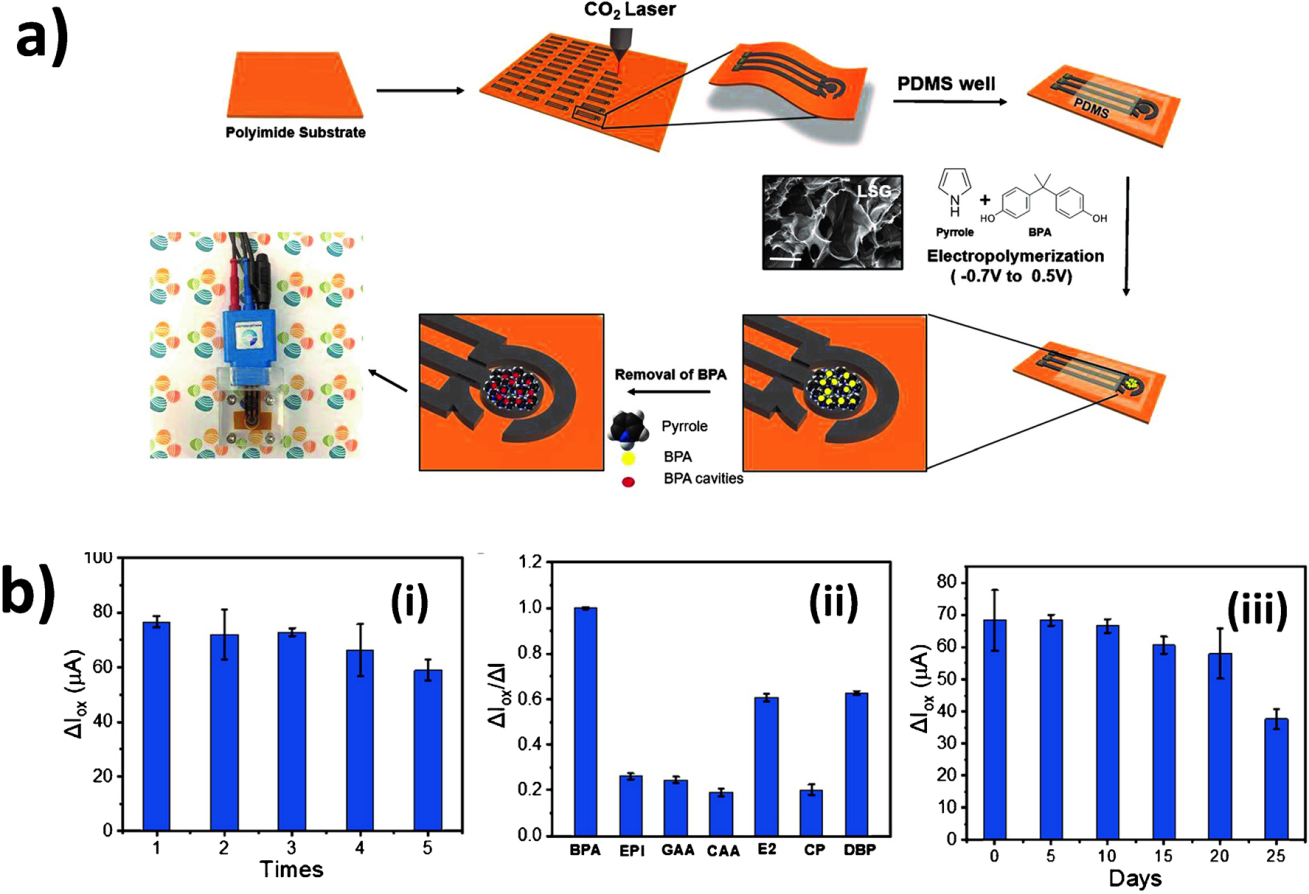
Molecular imprinted LIG. **a** The preparation process of molecular imprinted polymer (MIP) on LIG realized by polypyrrole (PPy) for BPA determination and the device integration. **b** Analytical performance with respect to reusability (i), selectivity (ii) by using 1 μM of BPA and other interferences (EPI: epinephrine GAA: gallic acid, CAA: caffeic acid, E2: estradiol, CP: chlorophenol, DBP: dibuthyl phatalathe), and stability (iii) [96]. **a** reprinted and **b** [96] adapted with permission from Elsevier

Molecular imprinting has become a highly promising technology to provide LIG electrodes with greater selectivity (Fig. 7) than bare electrodes as reported recently by Tutku et al. [96] who demonstrated molecularly imprinted bisphenol A (BPA) on LIG electrodes by using polypyrrole (PPy) as a polymer (Fig. 7a). The authors demonstrated the electrodes could be reused by washing with a solvent mixture (acetic acid and methanol) where a slight loss of signal responses was observed after being reused for four times (Fig. 7b i). The selectivity towards BPA was acceptable but still suffered from the interferences which share high similarity in the chemical structures (Fig. 7b i). A storage time of the electrodes up to 10 days without significant signal loss could be achieved (Fig. 7b iii).

The analytes and associated sensor validation parameters of all reviewed publications dealing with laser-induced carbon materials are summarized in Table 2 (Fig. 6) (Fig. 7).

**Table 2:** Summary of analytes detected with laser-generated carbon nanohybrids for non-enzymatic sensors

Analyte	Material	Method	Linear range	LOD	Reference
Glucose	CuO/LIG	Amperometry	1 μM–5 mM	0.1 μM	[39]
	LSG/Cu-NP	Amperometry	1 μM–4.54 mM	0.35 μM	[74]
	Cu NPs-LIG	Amperometry	1 μM–6 mM	0.39 μM	[91]
	Ni-LCNF	Amperometry	10–100 μM, 100 μM −5 mM	0.3 μM	[89]
	PDMS/AgNW/ LIG/PtAuNP	Amperometry	0–1.1 mM	5 μM	[97]
H_2_O_2_	PtLIG	Amperometry	0.5 μM–5 mM	0.2 μM	[92]
	LIG/AgNPs	Amperometry	0.1–10 mM	7.9 μM	[93]
Ascorbic acid	Pt/LIG	DPV	10–890 μM	6.1 μM	[75]
Uric acid			1–63 μM	0.22 μM	
Dopamine			0.5–56 μM	0.07 μM	
Bisphenol A	MIP/PPy@LIG	DPV	0.05–20 μM	8 nM	[96]

**Fig. 8:**
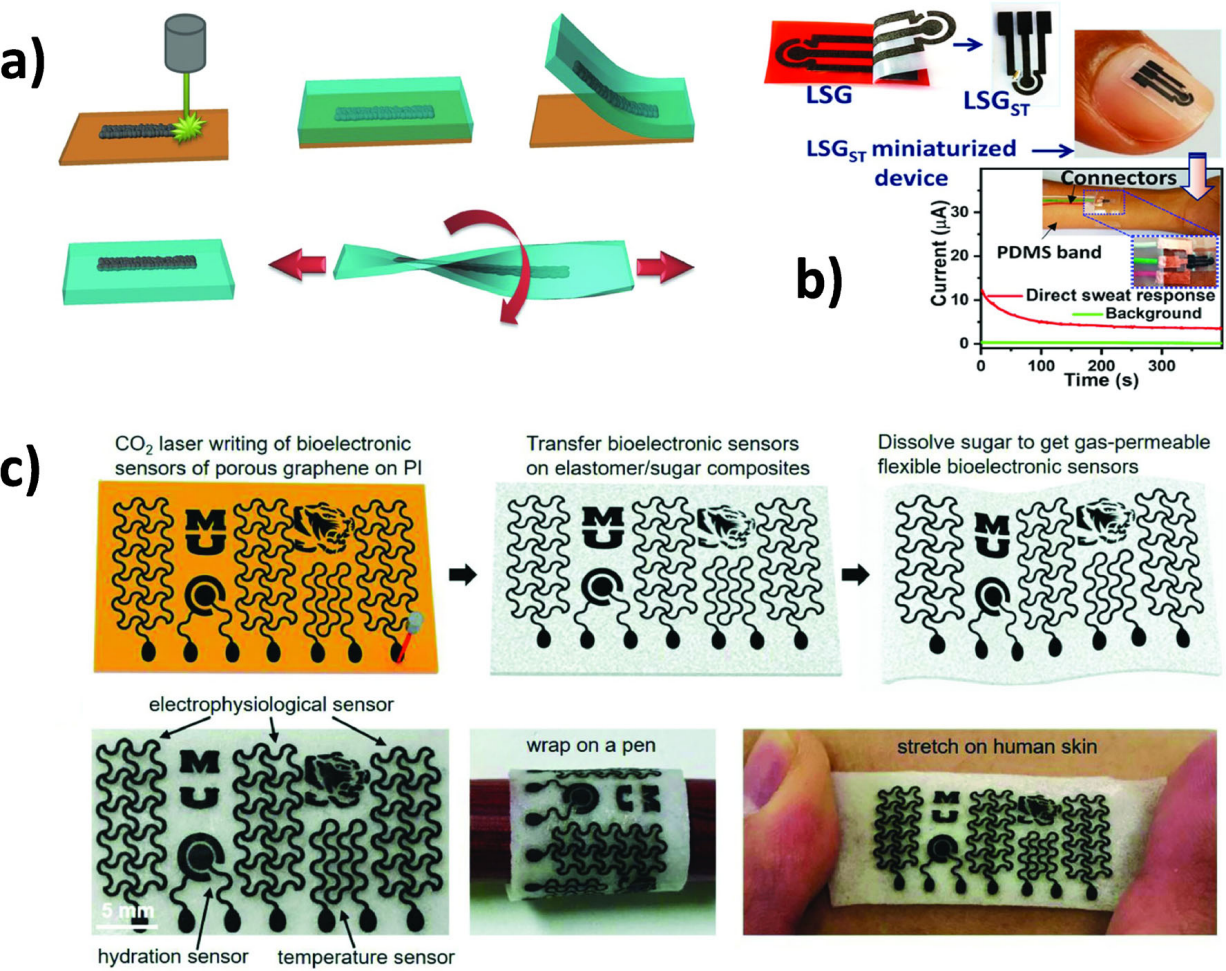
Wearable sensing systems based on laser-induced graphene. **a** Scheme of transferring LIG generated on polyimide foil (i) by casting with PDMS (ii) and subsequent peeling off (ii). The LIG is now bound to PDMS (iv) which offers elastic properties (v) [100]. **b** Transfer of LIG to Scotch tape that can be taped to the body and monitor analytes directly in produced sweat [39]. **c** Transfer of LIG to gas-permeable silicone elastomeric sponges (i), and multi-sensing abilities and demonstration of the flexible behavior of the sensor (ii) [101]. **a** [100] and **c** [101] adapted with permission from John Wiley & Sons, Inc. **b** adapted with permission from [39]. Copyright (2020) American Chemical Society

Future trends guide towards point-of-care sensors and wearable electronics due to the increasing awareness in health monitoring. Tao et al. [98] were among the first to report a wearable device based on LIG. They showed the functionality of LIG as an artificial throat which can generate and detect different sounds due to mechanical vibration and is, therefore, promising for disabled people [98]. Recently, Yang et al. [99] presented a lab-on-a-chip wearable patch completely created by laser writing. The sensor consists of multiple layers including a microfluidic module that allows dynamic sweat sampling while wearing and medical adhesive layers to mount the whole patch onto the skin. Additionally, the device is connected to a reusable flexible printed circuit board which makes wireless readout possible. They have verified in situ uric acid and tyrosine measurements and were able to correlate the obtained signals with healthy patients and patients with hyperuricemia or gout, making their lab-on-a-chip very useful for early diagnosis and prevention of this disease [99]. By simple casting with an elastomeric substrate, e.g., PDMS and peeling off, Lamberti et al. [100] demonstrated for the first time the transfer of LIG electrodes from polyimide onto flexible, transparent, and stretchable polymers (Fig. 8a). Similar approaches for transferring LIG to a wearable substrate were carried out by several other research groups. For example, Xuan et al. [97] decorated LIG with silver nanowires prior to PDMS casting to enhance the conductivity under mechanical deformations. After peeling off the PDMS composite, they electroplated Pt and Au NPs on the surface to enhance analyte sensing abilities. They demonstrated the direct detection of glucose in human sweat and proposed the application as wearable [97]. The direct sensing in secreted body sweat can be achieved by generating localized alkaline conditions at the sensor interface during measurements [131]. Prabhakaran and Nayak [39] modified their LIG by deposition of copper salt with subsequent treatment of focused sunlight to obtain crystalline Cu nanoparticles. By transferring the LIG to a Scotch brand tape, the electrode can be taped on the body (Fig. 8b). Before, NaOH solution was drop-casted and dried on the electrode surface whereby the alkaline conditions were retained. Therefore, they could directly detect glucose in sweat secreted on the skin without the need of injecting a sample into the NaOH matrix [39]. Sun et al. [101] realized the transfer of LIG to elastomeric silicone/sugar composites, which are also gas-permeable and can be comfortably worn on the skin and withstand multiple bending without losing sensing performance (Fig. 8c). Their prototype is able to monitor temperature and hydration changes and allows water permeation which reduces inflammation risks during long-time wearing [101]. The aim of the studies from Chhetry et al. [102] was the sensing of strain changes due to small movements on the human skin such as the wrist pulse. They hybridized LIG with MoS_2_ to achieve an electromechanical stability for over 12,000 strain/release cycles which is an idea that could also be adapted to improve wearable electrochemical sensors [102]. Another highly fascinating field is soft robotics that can interact with humans. In this regard, Ling et al. [103] fabricated LIG-based soft electrothermal actuators with several shapes that are capable of reconfigurable 3D assembly by mechanic guidance such as gesture control. Furthermore, the potential application as artificial muscle was investigated and the ability to lift a mass of about 110 times of the structure’s own weight was demonstrated. Their soft robotic finger is able to reversible wrap around a human finger on demand and carry out electrocardiogram measurements [103]. Those approaches can be well adapted for the development of wearable non-enzymatic sensors that can also interact with humans on demand.

Overall, carbon nanomaterial hybrids through laser writing have been obviously a promising candidate for implementing into the next-generation devices based on non-enzymatic electrochemical sensors. As can be seen in Table 3, they offer not only highly comparable performance to traditional carbon nanomaterials such as CNT and CNFs but also more attractive features in terms of cost and device fabrication. Many of previous studies in laser-generated carbon nanomaterial hybrids for other fields of applications, e.g., energy storage, could well facilitate the advancement of non-enzymatic electrochemical sensors that should be further explored.

**Table 3:** Comparison of analytical performance and fabrication of carbon nanomaterial hybrids for non-enzymatic sensors

Comparison aspects	Carbon nanotubes	Carbon nanofibers	Laser-induced graphene*
LOD of common analyte (M)			
Glucose	10^−8^ to 10^−5^ [104]	10^−7^** [89] 10^−7^ to 10^−6^*** [38]	10^−7^ to 10^−6^
H_2_O_2_	10^−8^ to 10^−5^ [104]	10^−5^ to 10^−7^ [38]	10^−7^ to 10^−6^
Dopamine	10^−10^ to 10^−9^ [105–107]	10^−7^ to 10^−8^ [38]	10^−8^
Uric acid	10^−7^ to 10^−6^ [108, 109]	10^−7^ [38]	10^−7^
Ascorbic acid	10^−9^ [110]	10^−4^ [38]	10^−6^
Complexity of instrumentation	High	Moderate	Low
Cost of material precursor	High	Moderate	Low
Complexity of device integration	High	High	Low

*See also Table 2; **CNFs from laser writing; ***CNFs from other synthesis methods

## Conclusion and outlook

High stability and low cost have brought non-enzymatic electrochemical sensors a great amount of research attention. They have long been developed, mostly, towards finding novel electrode materials that enable electrocatalytic reactions. A vast variety of nanomaterials based on metals, carbon, and derivatives have been successfully employed in the sensors with favorable analytical performance. In particular, using hybrids made of catalysts in a 3D-carbon nanomaterial scaffold, the sensitivity can be enhanced significantly. This is especially of interest for applications in microfluidic analytical systems where the number of analytes is limited. However, most previous reports are facing challenges associated with how to generate such high-performance transducers in a simple manner suitable for mass production. Introduction of the laser-induced carbonization strategy to generate graphene opens up a great opportunity to tackle these challenges. High flexibility in electrode designs and choice of substrate primarily facilitate the easy integration of high-performance non-enzymatic electrochemical transducers into miniaturized systems. Laser-generated carbon nanomaterials containing catalysts, e.g., with metal, metal oxide, and heteroatom-dopant, have been increasingly explored in the past few years, specifically in the energy-related fields. These developed materials, however, can be practically exploited in non-enzymatic electrochemical sensors which could become an interesting research topic in the very near future. Exploring more functionality and applicability of laser-induced nanocatalyst hybrids is highly encouraged.

With respect to the fabrication of transducers for non-enzymatic sensors, understanding the effect of laser on the as-generated electrocatalyts on an atomic level could give better control over their favorable functionalities. In addition, generating metallic nanocatalysts in the form of alloys with laser is interesting to investigate. With their fascinating morphological structures, electrospun nanofibers are considered a promising substrate. However, their successful carbonization is strongly dependent on the existence of metal within the fibers. The development of metal-free laser-induced carbon nanofibers is of interest for many biomedical applications where cytotoxicity is a primary concern. Carbonaceous nanomaterials such as graphene and carbon nanotubes could be a potential candidate to promote efficient heat dissipation of nanofibers. This is specifically important for the development of heteroatom-doped carbon nanofibers. Furthermore, in order to create a greater resolution of the non-enzymatic transducer, other laser types with smaller wavelengths should be investigated. Lastly, similar to other fabricated electrochemical sensors it is necessary to investigate the long-term stability of the laser-generated electrodes as well as proper storage conditions.

There are some critical issues that need to be overcome before non-enzymatic electrochemical sensors (made either through conventional fabrication or laser writing) can become powerful tools to actually substitute conventional enzyme-based sensors. The selectivity of non-enzymatic sensors is still very poor. In this case, molecular imprinting can be an efficient strategy to cope with the problem [96, 111]. In addition to this aspect, many metal oxide electrocatalysts strictly require alkaline conditions which make on-site, real-time, and in vivo monitoring troublesome. In situ locally generated alkaline environment at the electrodes is a promising approach as demonstrated by Prabhakaran and Nayak [39], and Strakosas et al. [112]. The effect of specific sample matrices on the generated electrocatalytic current should be thoroughly investigated to enable sample-to-answer analytical devices. Establishing robust integration methods of non-enzymatic electrochemical transducers to miniaturized devices will eventually enable the point-of-care testing device with high performance as well as high affordability.
